# The complete mitochondrial genome sequence of big-eyed Mountain keelback *Pseudoxenodon macrops*

**DOI:** 10.1080/23802359.2020.1715284

**Published:** 2020-01-20

**Authors:** Jiahong Liao, Min Tang, Liqing Peng, Lichun Jiang, Zhangqiang You, Wei Chen

**Affiliations:** Ecological Security and Protection Key Laboratory of Sichuan Province, Mianyang Normal University, Mianyang, China

**Keywords:** Colubridae, mitochondrial genome, *Pseudoxenodon macrops*

## Abstract

In this study, the complete mitochondrial genome of big-eyed mountain keelback *Pseudoxenodon macrops* was sequenced adopting Illumina high-throughput sequencing technology. The complete mitogenome of the species was 19,444 bp in length, including 13 protein-coding genes, 22 transfer RNA (tRNA) genes, two ribosomal RNA genes, and two non-coding control regions (CR). The overall base composition of mitogenome was 32.0% A, 25.5% T, 28.2% C, and 14.3% G. Most mitochondrial genes are encoded on the heavy strand, only ND6 and eight tRNA genes are on the light strand. We expect that the presented mitogenome can provide important data for future studies on phylogenetic relationship and population genetics of this species.

Big-eyed mountain keelback *Pseudoxenodon macrops* is a species of snake in the family Colubridae and found in India, Nepal, Myanmar, Thailand, Vietnam, and China (Zhao 2006). It mainly inhabits in mountains with an altitude of above 800 m (Zhao and Adler 1993; Zhao 2006). In this study, we used Illumina high-throughput sequencing technology to sequence the whole mitogenomic region of this species. The specimen was collected at roadside from Chunyang Palace (103°25′3.76″E, 29°34′2.05″N, 951 m) of Emei Mountain Sichuan province, China, and the specimen is stored in the museum of college of life science and biotechnology Mianyang Normal University (specimen code MNU20190612).

The length of the complete mitochondrial genome of *P. macrops* is 19,444 bp with the GenBank accession No. MN411631.1, including 13 protein-coding genes, 22 transfer RNA (tRNA) genes, two ribosomal RNA (rRNA) genes, and two control regions. The overall base composition is A: 32.0%, T: 25.5%, C: 28.2%, and G: 14.3%, with an A + T bias (57.5%). Only ND6 and eight tRNA genes are encoded on the light strand, and all the other genes on the heavy strand. Among all 13 mitochondrial protein-coding genes, ND2, ND3, COXII, ATP8, ATP6, ND4L, ND5, ND6, and Cytb share the start codon ATG; ND1 and ND4 have the start codon ATA; COXI and ND4 show different start codons (GTG and ATA, respectively). Five protein-coding genes (ND1, ND2, ND3, COXII, and COXIII) are inferred to terminate with an incomplete stop codon T––; five protein-coding genes (ATP8, ATP6, ND4L, ND5, and CYTB) share the typical termination codon TAA; COXI and ND6 have stop codon AGA, and ND4 use AGG as a stop codon. For 13 protein-coding genes, the A + T content ranges from 52.58% to 61.73%, which is higher than that of G + C (Jablonski et al. 2019). In addition, we detected that 18 nucleotides from nine locations overlapped with the length of overlapped sequence of 1–5 bp, whereas there are totally 51 bp intergenic nucleotides in seven locations with the length of intergenic spacer of 1–33 bp. Twenty-two pairs of genes are directly adjacent without intergenic or overlapping nucleotides. 12S rRNA (916 bp) and 16S rRNA (1483 bp) are located between tRNA-Phe and ND1 and separated by tRNA-Val gene, and the lengths of 22 tRNA genes range from 56 to 74 bp. The two non-coding regions, one of the putative CR (2587 bp in length), were bound by tRNA-Ile and tRNA-Leu and the other (1721 bp in length) surrounded by tRN-Pro and tRN-Phe. A NJ phylogenetic tree of complete mitochondrial genomes analyses of snakes of Colubridae showed that *P. macrops* did not have close relationship with Colubridae species (Figure 1).

**Figure 1.**
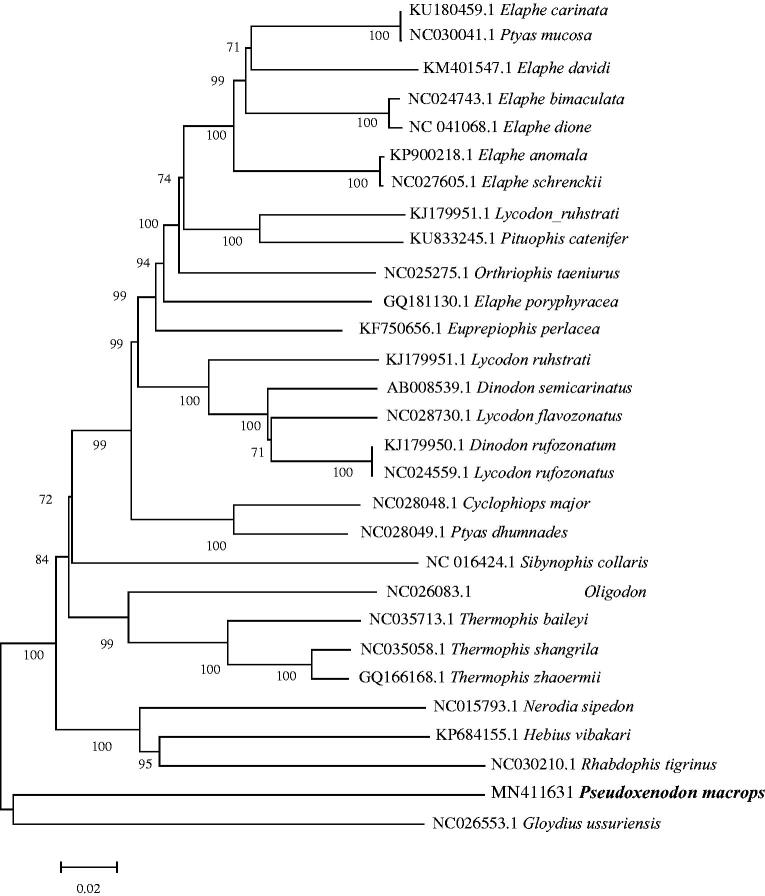
Neighbour-Joining phylogenetic tree of Colubridae representatives produced based on complete mitochondrial genomes. Genbank accession numbers and bootstrap values of nodes are shown on the tree.

In this study, the whole nucleotide sequence of the *P. macrops* mitogenome was explored and these basic data will contribute to further the phylogenetic relationships and population genetics analysis of genus *Pseudoxenodon* in the future.
